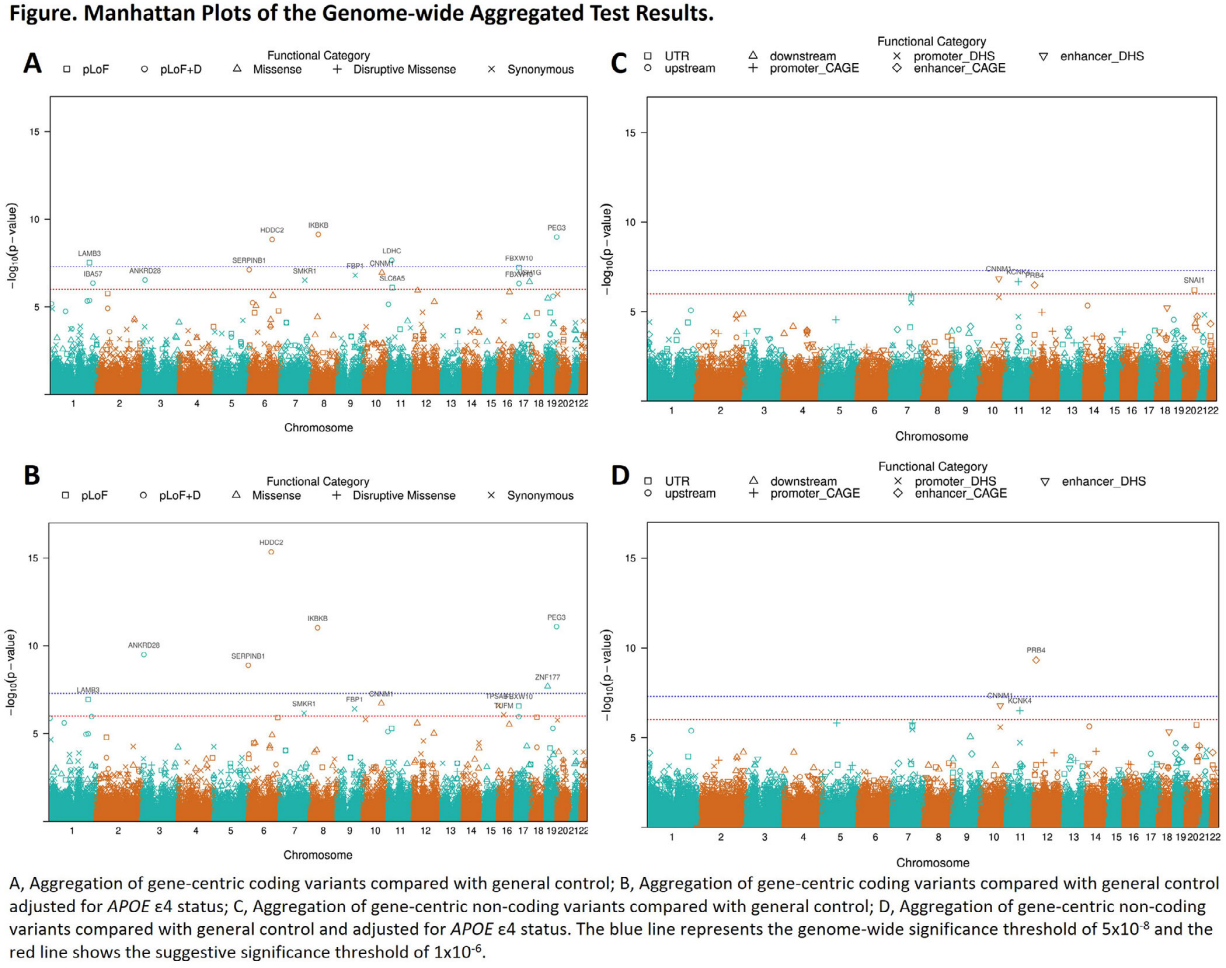# Whole Genome Sequencing Analysis of Cognitively Wellderly Individuals Identifies Potential Protective Genetic Variants for Alzheimer’s Disease

**DOI:** 10.1002/alz.088478

**Published:** 2025-01-03

**Authors:** Dongyu Wang, Seung Hoan Choi, Sabrina Abbruzzese, Morgan A Rosser, Joshua C Bis, Myriam Fornage, Eric Boerwinkle, Claudia L Satizabal, Bruce M. Psaty, Oscar L. Lopez, Thomas H. Mosley, Yanbing Wang, Josée Dupuis, Anita L. DeStefano, Sudha Seshadri, Gina M. Peloso

**Affiliations:** ^1^ Boston University School of Public Health, Boston, MA USA; ^2^ University of Washington, Seattle, WA USA; ^3^ Human Genetics Center, School of Public Health, University of Texas Health Science Center, Houston, TX USA; ^4^ The University of Texas Health Science Center at Houston, Houston, TX USA; ^5^ University of Texas Health Science Center at Houston, Houston, TX USA; ^6^ Brown Foundation Institute of Molecular Medicine, McGovern Medical School; School of Public Health, The University of Texas Health Science Center, Houston, TX USA; ^7^ University of Texas Health Science Center at Houston School of Public Health, Houston, TX USA; ^8^ The University of Texas Health Science Center at San Antonio, San Antonio, TX USA; ^9^ Glenn Biggs Institute for Alzheimer’s & Neurodegenerative Diseases, University of Texas Health Science Center at San Antonio, San Antonio, TX USA; ^10^ University of Pittsburgh, Pittsburgh, PA USA; ^11^ University of Pittsburgh Alzheimer’s Disease Research Center, Pittsburgh, PA USA; ^12^ University of Mississippi Medical Center, Jackson, MS USA; ^13^ McGill University, School of Population and Global Health, Montreal, QC Canada; ^14^ The National Heart, Lung, and Blood Institute’s Framingham Heart Study, Framingham, MA USA; ^15^ Framingham Heart Study, NHLBI, Framingham, MA USA; ^16^ Glenn Biggs Institute for Alzheimer’s & Neurodegenerative Diseases, University of Texas Health Sciences Center at San Antonio, San Antonio, TX USA; ^17^ Department of Neurology, University of Texas Health Sciences Center, San Antonio, TX USA

## Abstract

**Background:**

Genetic variants that confer protection from Alzheimer’s disease (AD) may be particularly critical in developing therapeutics. To target protective variant identification, we performed genetic association testing among selected individuals with whole genome sequencing (WGS) that remained alive and dementia‐free beyond age 85 (“Wellderly”).

**Methods:**

We selected 1,873 White and Black Wellderly individuals with documented normal cognition beyond age 85 as determined by direct, in‐person assessment with WGS from the NHLBI TOPMed project. We used two sets of comparison groups: (i) general controls, non‐Wellderly individuals including persons without and with dementia [n = 8,502] and (ii) individuals who developed dementia before age 85 [n = 810]. We performed generalized mixed model regression for each variant with a minor allele frequency (MAF) > 1%, as well as analysis of aggregation of rare (MAF ≤ 1%) coding and non‐coding variants via a modified STAAR approach, with and without adjusting for *APOE* status.

**Results:**

We observed a reduced likelihood of Wellderly status compared to general controls at the *APOE* locus (rs429358, MAF = 14%, odds ratio [OR] = 0.69, p = 1.6 × 10^‐10^), consistent with the known association of the *APOE* locus with dementia. We also observed 7 coding gene‐based tests associated with Wellderly status at an alpha of 5 × 10^‐8^ (**Figure**). For the aggregation of non‐coding variants, we observed Wellderly status associated with *PRB4* (p = 4.7 × 10^‐10^) and suggestively (p<1 × 10^‐6^) associated with 3 additional genes. None of the variant aggregates showed significant association with Wellderly status when compared to the diseased controls. *IKBKB* encodes an inhibitor of nuclear factor kappa B kinase subunit beta, and deficiency of IKBKB protein in myeloid cells was reported to have improved cognitive functions in the AD mice. *KCNK4*, the potassium channel subfamily K member 4, plays an important role in controlling the cell potassium flux and dysregulated *KCNK4* function can cause neurodevelopmental abnormalities.

**Conclusion:**

Although only variants in the *APOE* locus have a reduced likelihood of Wellderly status in the single variant analysis, our aggregated tests suggest genes aside from *APOE* that are associated with Wellderly status with biological plausibility. We are pursuing replication using the Alzheimer’s disease Sequencing Project (ADSP) WGS data.